# Treatment of osteoporosis with teriparatide: The Slovenian experience

**DOI:** 10.1515/med-2021-0359

**Published:** 2021-10-15

**Authors:** Tomaz Kocjan, Antonela Sabati Rajic, Mojca Jensterle Sever, Andrej Janez, Gaj Vidmar, Nina Orehek, Janja Marc, Barbara Ostanek

**Affiliations:** Department of Internal Medicine, Faculty of Medicine, University of Ljubljana, Zaloška cesta 007, Ljubljana, 1000, Slovenia; Department of Endocrinology, Diabetes and Metabolic Diseases, University Medical Centre Ljubljana, Ljubljana, Slovenia; Department of Internal Medicine, Faculty of Medicine, University of Ljubljana, Ljubljana, Slovenia; Department of Biostatistics and Scientific Informatics, University Rehabilitation Institute, Ljubljana, Slovenia; Department of Psychology, FAMNIT, University of Primorska, Koper, Slovenia; Department of Clinical Biochemistry, Faculty of Pharmacy, University of Ljubljana, Ljubljana, Slovenia; Department of Biostatistics and Medical Informatics, Faculty of Medicine, University of Ljubljana, Ljubljana, Slovenia

**Keywords:** bisphosphonates, bone mineral density, osteoporosis, teriparatide

## Abstract

The aim of this study was to investigate the characteristics of postmenopausal women prescribed with teriparatide in Slovenia, during the first decade after its approval, and the predictors of bone mineral density (BMD) improvement with treatment. We retrospectively studied postmenopausal osteoporotic patients prescribed with teriparatide at tertiary center from 2006 to 2015. BMD was measured at standard sites by DXA at baseline, after 12 and 24 months. 25-hydroxyvitamin D and procollagen type I N-terminal propeptide (PINP) were measured at the same time-points. The inclusion criteria were met by 188 women (aged 71 years on average), 151 (80.3%) with postmenopausal and 37 (19.7%) with glucocorticoid-induced osteoporosis. Everyone had at least one fracture, 159 (84.6%) had ≥2 fractures, with vertebral fractures in 172 patients (91.5%). All patients had been previously on antiresorptives for 8.6 years on average. The average BMD change at lumbar spine, total hip, and femoral neck was +5.0%, −1.1%, and +0.3% after 24 months of treatment, respectively. Higher baseline PINP was associated with higher BMD increase at all sites after the first 12 months. Teriparatide was prescribed mostly to elderly women with severe osteoporosis who had sustained two or more fractures despite long-term antiresorptive therapy. Baseline PINP might predict initial BMD increase with teriparatide.

## 1 Introduction

Osteoporosis is a systemic skeletal disease characterized by increased bone fragility, which affects mainly postmenopausal women and represents a substantial public health problem [1]. Approximately 16,000 osteoporotic fractures were sustained in Slovenia in 2010 with the ensuing economic burden estimated at €56 million. Further increase in both the number of fractures and costs was projected for 2025 due to aging and considerable treatment gap in high-risk population [2].

Potent antiresorptive agents such as bisphosphonates and denosumab combined with vitamin D and calcium are efficacious against all types of osteoporotic fractures [3]. However, antiresorptive therapy is not able to rebuild bone that has been lost due to increased remodeling after menopause. Osteoanabolic drugs are an attractive alternative because they directly stimulate bone formation and improve microarchitecture of the skeleton [4]. In Slovenia, teriparatide, a fully active human recombinant fragment of parathyroid hormone (PTH_1–34_), represents the only osteoanabolic drug for osteoporosis at present. A randomized trial proved its efficacy against vertebral and non-vertebral fractures [5]. Bone mineral density (BMD), a major surrogate endpoint for fractures, was also shown to improve with teriparatide [5]. Another option for monitoring the treatment is with a bone formation marker such as procollagen type I N-terminal propeptide (PINP), which increases few months after teriparatide initiation [6].

Despite being limited to two years once in a lifetime, treatment with teriparatide is associated with significant cost, so it is usually reserved for high-risk patients with severe osteoporosis as a second-line treatment [3]. Still, the reimbursement policies world-wide are extremely variable [7]. According to Slovenian guidelines, every candidate for teriparatide should be discussed and agreed at the medical council for osteoanabolic treatment of osteoporosis, which was initially founded at University Medical Centre Ljubljana in 2006 and then at University Medical Centre Maribor in 2011. Only patients with primary or glucocorticoid-induced osteoporosis (GIOP) who sustained a vertebral, hip, or proximal humerus fracture after at least a year of prior treatment with antiresorptives could receive teriparatide [8].

Additionally, daily subcutaneous injections present significant burden to patients who might also experience response failure to teriparatide, defined as an insufficient BMD increase less than 3% from baseline [9]. Early attempts to identify predictors of treatment failure were inconclusive [9], but some of more recent reports exposed prior treatment with bisphosphonates as one of the main possible reasons [10,11].

We aimed to analyze the characteristics of postmenopausal women prescribed with teriparatide according to Slovenian guidelines during the first decade after its approval in 2006. We also tried to identify the predictors of BMD improvement with this treatment to better select future candidates who would benefit from teriparatide.

## 2 Methods

### 2.1 Study design

**Figure 1:**
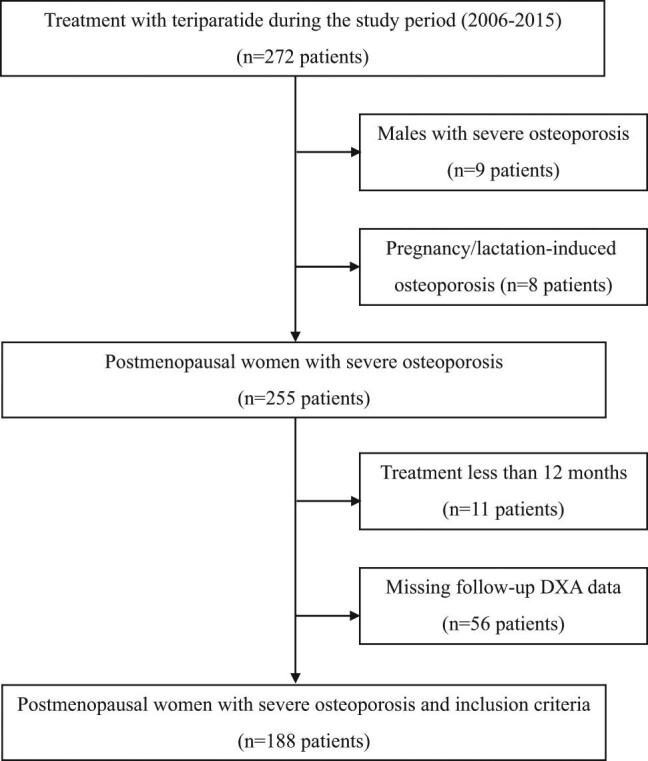
Study design. Notes. DXA – dual-energy X-ray absorptiometry.


A retrospective study was conducted using data from the period 2006–2015 collected at the national tertiary endocrine center, which is the main (and the only one until 2011) medical facility in Slovenia with availability of osteoanabolic treatment. Eligible patients for inclusion were postmenopausal women who had received teriparatide (20 µg once daily by subcutaneous injection) for at least 12 months and had at least two available BMD measurements at our institution (Figure 1).

**Ethics approval:** The data collection and its analysis were approved by the Republic of Slovenia National Medical Ethics Committee, ID 152/03/09.
**Informed consent:** Informed consent was obtained from all individual participants included in the study.

### 2.2 Clinical assessment

Details of each patient’s medical history with prior fractures and types of previous antiresorptive agents were recorded. Clinical examination and extended laboratory tests with bone turnover markers were performed to exclude systemic and metabolic bone diseases other than postmenopausal osteoporosis or GIOP [8]. All patients were trained to use the proper injection techniques and educated about importance of osteoanabolic treatment by our dedicated endocrine nurses. They were also prescribed with vitamin D3 of 1,000 IU daily and were instructed to ingest 1,200 mg of calcium daily. Regular clinical follow-ups with routine laboratory checkups were scheduled at every six months during treatment with teriparatide at our outpatient clinic. BMD measurements at the lumbar spine (LS), total hip (TH), and femoral neck (FN) were performed by DXA (Discovery, Hologic, USA) at baseline, after 12 months of treatment, and when teriparatide was stopped. PINP and 25-hydroxyvitamin D were measured at the same time-points. Radiographs were obtained if subjects had symptoms suggestive of a new clinical osteoporotic fracture. Serum 25-hydroxyvitamin D levels and serum TSH levels were determined with competitive chemiluminescent immunoassay and with immunometric test, respectively. Serum levels of intact PTH, PINP, and C-terminal cross-linked telopeptide (CTX) as a marker of bone resorption were determined with chemiluminescent immunometric assay. Measurements were done at our clinical laboratory using routine quality control procedures.

### 2.3 Statistical analysis

Descriptive statistics were calculated for all variables. Increase in BMD after 12 months and from 12 to 24 months was modeled using multiple linear regression for the patients who completed the treatment (*n* = 168). Potential predictors were selected based on clinical consideration and data quality and entered together into the model.

Regression diagnostic plots (histograms and normal p–p plots of residuals and scatterplots of standardized residuals against predicted value) showed no marked violations of the assumptions (normal distribution of residuals and homoscedasticity). Regression diagnostic statistics did not indicate problems with collinearity of predictors (tolerance values were above 0.4 and variance inflation factor values were under 3).

Each regression model was fitted in two ways: first by excluding all the patients with a missing value of any of the predictors (complete case analysis, a.k.a. list-wise deletion of missing data) and then by replacing all the missing values with mean value of respective predictors (mean imputation). The predictors that turned out to be statistically significant in both analyses can be considered more reliable. Two measures of model fit are reported for each model: *p*-value for statistical significance of the model as a whole (from *F*-test) and adjusted *R*-squared (estimated proportion of explained variation in the population). Statistical significance level was set at *p* ≤ 0.05. Statistical analyses were conducted using IBM SPSS Statistics 25 (IBM Corp., Armonk, USA, 2017).

## 3 Results

### 3.1 Baseline patient characteristics

The inclusion criteria were met by 188 females (Figure 1), aged 71 on average (range 49–87 years); 151 (80.3%) with postmenopausal osteoporosis and 37 (19.7%) with GIOP. Everyone had severe osteoporosis, mostly (159, 84.6%) with two osteoporotic fractures or more. Vertebral fractures were present in 172 patients (91.5%) and non-vertebral fractures in the rest. Everybody had been previously on long-term osteoporosis treatment for 8.6 years on average. Directly before teriparatide, 138 patients (73.4%) received a bisphosphonate, 36 patients (19.1%) strontium ranelate, 7 (3.7%) denosumab, and 7 (3.7%) raloxifene. Clinical characteristics and laboratory parameters of the study group are presented in Table 1.

**Table 1:** Patients’ clinical and biochemical characteristics

Characteristic	Mean value (SD)	Median (range)
Age (years)	71.0 (7.9)	72.0 (49.0, 87.0)
Menopause duration (years)	22.9 (9.1)	24.0 (4.0, 51.0)
Previous treatment duration (months)	103.3 (49.4)	102.0 (7.0, 266.0)
BMI (kg/m^2^)	26.3 (4.2)	25.8 (18.7, 41.3)
Corrected serum calcium (mmol/L)	2.2 (0.1)	2.2 (2.0, 2.6)
Urinary calcium (mmol/L)	2.8 (1.9)	2.4 (0.2, 8.4)
eGFR (mL/min)	75.0 (19.3)	76.0 (27.0, 100.0)
Serum urate (μmol/L)	275.8 (78.8)	267.0 (109.0, 599.0)
TSH (mIU/L)	2.0 (4.9)	1.3 (0.4, 6.5)
Intact PTH (ng/L)	46.9 (22.1)	42.9 (9.9, 112.0)
25-Hydroxyvitamin D (nmol/L)	65.6 (24.2)	66.5 (10.0, 125.0)

Characteristic	Category	Number (Proportion)
Category of osteoporosis	Postmenopausal	151 (80.3%)
	GIOP	37 (19.7%)
Comorbidities*	Yes	80 (42.6%)
	No	108 (57.4%)
Calcium supplements	Yes	122 (64.9%)
	No	66 (35.1%)
Vitamin D supplements	Yes	174 (92.6%)
	No	14 (7.4%)
25-Hydroxyvitamin D category**	<50 nmol/L	47 (25.5%)
	50–75 nmol/L	68 (37%)
	>75 nmol/L	69 (37.5%)
Vertebral fractures	Yes	172 (91.5%)
	No	16 (8.5%)
Osteoporotic fractures (number)	1	29 (15.4%)
	2	24 (12.8%)
	>2	135 (71.8%)
Smoking	Never	143 (76.1%)
	Past	34 (18.1%)
	Current	11 (5.9%)

Numerical variables are described as mean value (SD) and median (range); categorical variables are reported as frequencies and proportions.

Notes: *e.g., Rheumatoid arthritis, cardiovascular disease, COPD, type 2 diabetes, dementia, Parkinson’s disease; **<50 nmol/L – insufficient, 50–75 nmol/L – borderline; >75 nmol/L – sufficient; eGFR – estimated glomerular filtration rate; GIOP – glucocorticoid-induced osteoporosis.

### 3.2 Safety and duration of treatment

Transient hypercalciuria was detected in 10% and hypercalcemia in 2% of the patients. Other adverse events noted by 31 (16.5%) patients were dizziness (18; 9.6%), bone pain (9; 4.8%), nausea (8; 4.3%), leg cramps (6; 3.2%), myalgias (6; 3.2%), and headache (2; 1.1%). The average duration of treatment with teriparatide was 22.5 (4.6) months. Twenty patients (10.6%) did not finish the treatment regimen (9 due to adverse events and 11 without obvious reason).

### 3.3 Bone related parameters before and after treatment

Patients’ BMD values with T-scores at baseline and BMD changes after treatment are presented together with bone turnover markers in Table 2. The average BMD change at LS, TH, and FN was +3.9%, −1.1%, and −1.2% during the first 12 months of treatment, respectively. During the subsequent 12 months, BMD at LS and FN increased by 1.1 and 1.5% on average, respectively, while BMD at TH remained stable on average. One patient sustained a proximal humerus and distal radius fracture, while 10 patients experienced one new fracture (two clinical vertebral and eight non-vertebral; no hip fractures) during treatment.

**Table 2:** Patients’ BMD and bone turnover markers at baseline with the changes after 12 or 24 months of treatment; variables are described as mean value (SD) and median (range)

Characteristic	Mean value (SD)	Median (range)
LS BMD (g/cm^2^)	0.785 (0.159)	0.772 (0.406, 1.357)
LS T-score (SD)	−2.381 (1.445)	−2.500 (−5.830, 2.820)
TH BMD (g/cm^2^)	0.721 (0.123)	0.713 (0.343, 1.068)
TH T-score (SD)	−1.969 (1.096)	−2.045 (−5.350, 1.130)
FN BMD (g/cm^2^)	0.595 (0.107)	0.591 (0.332, 0.870)
FN T-score (SD)	−2.287 (0.962)	−2.329 (−4.660, 0.190)
Δ LS in first 12 months (g/cm^2^)	0.031 (0.048)	0.026 (−0.158, 0.159)
Δ LS in second 12 months (g/cm^2^)	0.009 (0.048)	0.016 (−0.238, 0.121)
Δ TH in first 12 months (g/cm^2^)	−0.008 (0.035)	−0.008 (−0.116, 0.178)
Δ TH in second 12 months (g/cm^2^)	0.001 (0.029)	0.002 (−0.144, 0.079)
Δ FN in first 12 months (g/cm^2^)	−0.007 (0.041)	−0.009 (−0.148, 0.164)
Δ FN in second 12 months (g/cm^2^)	0.009 (0.042)	0.011 (−0.125, 0.229)
CTX (pg/mL)*	363.4 (269.3)	305.8 (10.0, 380.6)
PINP (μg/L)*	35.3 (19.2)	31.8 (7.1, 119.9)
PINP after 12 months (μg/L)	140.6 (88.9)	118.4 (6.0, 481.6)
PINP after 24 months (μg/L)	104.2 (69.7)	83.8 (12.4, 492.1)

Notes: LS – lumbar spine; TH – total hip; FN – femoral neck; CTX – C-terminal cross-linked telopeptide; PINP – procollagen type I N-terminal propeptide.

*Normal range for postmenopausal women: CTX 141.9–1,350 pg/mL; PINP 27.7–127.6 μg/L.

### 3.4 Predictors of BMD increase during treatment

The results of the multiple linear regression models are summarized in Tables 3 and 4. For the first 12 months (Table 3), the clearest finding was that higher PINP was associated with higher BMD increase at all sites. In addition, higher eGFR and higher urate concentration were (possibly) associated with higher BMD increase at LS, while normal vitamin D level, higher intact PTH, previous treatment with bisphosphonates, and higher number of fractures were (possibly) weakly associated with higher BMD increase at TH.

**Table 3:** Summary of multiple linear regression models for predicting increase in BMD in the first 12 months (*n* = 168 patients who had completed the treatment)

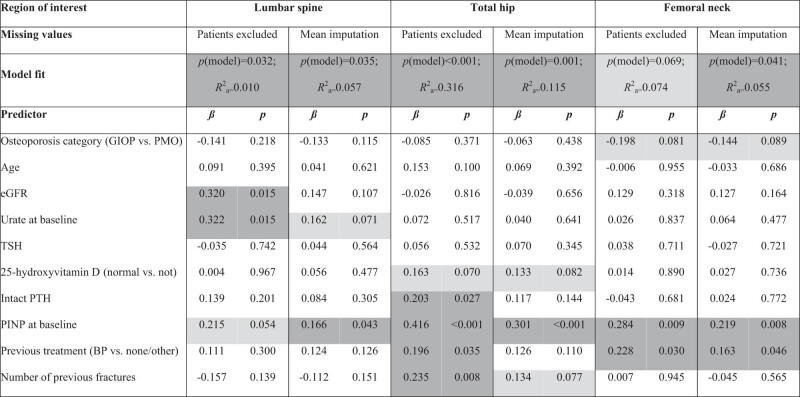

Notes: Predictors measured at the start of the study; *R* ^2^_a_ – adjusted coefficient of determination, *ß* – standardized regression coefficient; model fit measures (*p*-value and *R* ^2^_a_) are shaded in dark grey for the models with *p* ≤ 0.05 and light grey for models with 0.05 < *p* ≤ 0.10; within those models, standardized regression coefficients and *p*-values are shaded the same way). GIOP – glucocorticoid induced osteoporosis; PMO – postmenopausal osteoporosis; eGFR – estimated glomerular filtration rate; PINP – procollagen type 1 N-terminal propeptide; BP – bisphosphonate; no units are listed because only standardized regression coefficients are reported.

**Table 4:** Summary of multiple linear regression models for predicting increase in BMD from 12 to 24 months (*n* = 168 patients who completed the treatment)

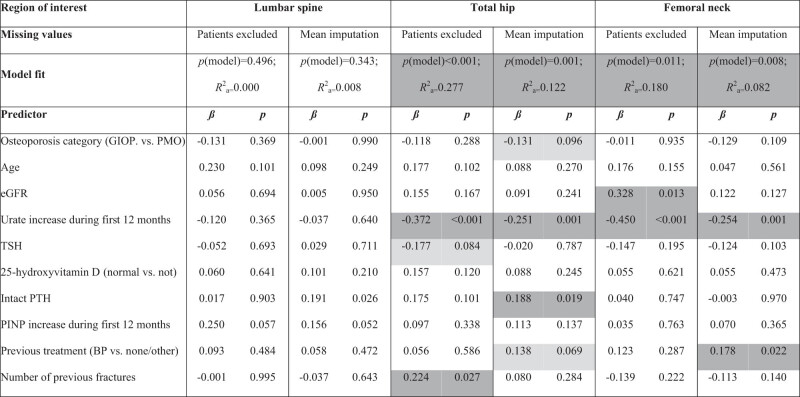

Notes: Predictors measured at the start of the study unless indicated otherwise; *R* ^2^_a_ – adjusted coefficient of determination, *ß* – standardized regression coefficient; model fit measures (*p*-value and *R* ^2^_a_) are shaded in dark grey for the adequate models and light grey for the marginally adequate models; within those models, standardized regression coefficients and *p*-values are shaded the same way). GIOP – glucocorticoid induced osteoporosis; PMO – postmenopausal osteoporosis; eGFR – estimated glomerular filtration rate; PINP – procollagen type 1 N-terminal propeptide; BP – bisphosphonate; no units are listed because only standardized regression coefficients are reported.

Between 12 and 24 months (Table 4), increase in BMD at LS could not be statistically significantly predicted. At TH and FN, higher urate increase during the first 12 months was associated with lower BMD increase. No other statistically significant predictor was reliably confirmed (i.e., in the complete case analysis as well as with mean imputation of missing data) at those two sites.

## 4 Discussion

In the first decade after approval in Slovenia, teriparatide was prescribed mostly to elderly women with severe osteoporosis. Over 84% of them had already sustained at least two fractures, a notably higher proportion than in the multinational European and Asian cohorts where only 65% [12] and 40% [13] of teriparatide initiators, respectively, were equally affected. Furthermore, 90% of our patients, 78% of European patients [12], and only 33% of Asian patients [13] had a history of a vertebral fracture. Everyone in our cohort had at least one fracture at baseline and had been on other osteoporosis medications before initiating osteoanabolic therapy. These observations simply reflect restrictive Slovenian guidelines [8]; however, the same approach was also advocated by the existing European guidance [3]. Contrary to our prescription policy, almost 15% of the European cohort had not sustained a fracture and more than 10% of them were treatment naïve [12]. The difference was even more striking in the Asian patients, where approximately one third was without fractures and never treated before [13], most probably due to more generous reimbursement [7].

Interestingly, the average T-scores of our patients at all sites were in the osteopenic range, which underscores the well-known imperfect capacity of BMD for fracture prediction [14]. In addition, around 20% of the cohort had GIOP, where fractures occur at higher T-scores [15]. Previous long-term antiresorptive treatment could have improved BMD [3]. Prevalent vertebral fractures might have also spuriously increased BMD at LS and the respective T-scores [16]. Some of our patients experienced new osteoporotic fractures despite osteoanabolic treatment. In the seminal randomized trial, teriparatide in patients with previous vertebral fractures decreased the fracture risk by up to 90%, but it did not abolish the fracture risk completely [5]. Furthermore, all our patients were at imminent fracture risk after previous recent major fractures [17] that allowed prescription of teriparatide [8]. The high persistence rate of almost 90% in our cohort could have been attributed to careful selection of patients and severity of their osteoporosis together with regular follow-ups and education of patients by our dedicated nurses, which proved valuable also in other observations [18]. Adverse events and their prevalence resembled the previously described experience with teriparatide [5].

The mean BMD gain in our patients was lower than originally reported at all sites [5]. Severity or extended duration of osteoporosis might have contributed to this finding [11]. The average duration of osteoporosis treatment before teriparatide in our cohort was more than eight and half years, and almost three quarters of patients received bisphosphonates during this period. Prior bisphosphonate treatment predicted BMD response failure with teriparatide in some [10,11], but not in all studies [19,20]. We were not able to directly confirm the negative impact of bisphosphonates and there was even an inconsistent signal to the opposite. This was likely a chance finding, as higher baseline PINP predicted higher BMD increase in our patients at all sites during the first year of teriparatide. Suppressed bone turnover with lower PINP values is a hallmark of long-term treatment with bisphosphonates [21]. Therefore, not only the exposure to bisphosphonates but the exact duration of treatment also might have better predicted the BMD change [10]. Our findings of higher PINP as predictor of better BMD gain are concordant with the teriparatide study by Chen et al. [22] and also with findings by Bauer et al. who examined the effects of PTH_1–84_ [23]. However, in these two studies, stronger and more consistent associations were observed between BMD gain and the 1- and 3-month changes in PINP [22,23], which we were unable to test, because bone turnover markers were not determined at these time-points. In our model, PINP increase during the first 12 months was not associated with BMD change in the second year of treatment. Similarly, Blumsohn et al. found only weak correlation of PINP increase during the first 6 months and BMD gain after 24 months at LS, but not at TH and FN [24]. In addition, we found higher eGFR and higher serum urate to be probably associated with higher BMD increase at LS during the first year of treatment. While skeletal responsiveness to PTH is reduced in patients with renal failure [25], serum urate had recently been associated with increased BMD [26] and a positive relationship between the change in serum urate and change in BMD with thiazides was reported [27]. Teriparatide was also linked to increased incidence of hyperuricemia [5], but contrary to the thiazide effects [27], higher urate increase during the first 12 months in our cohort predicted lower BMD increase in the second year of treatment.

Our study has some limitations. The design was retrospective and exclusion of all patients without at least two available sets of BMD data might have caused patient-selection bias. However, the assessment of our patients was consistent throughout the study. The criteria for the initiation of teriparatide remained unchanged and they were strictly followed in all cases. Data on previous osteoporosis medications should have ideally included exact duration of treatment for each drug, but because our patients were previously treated by different physicians from all over the country, who referred them to us for teriparatide treatment, the available data on previous treatment duration were of low quality.

The main strength of our study is that we studied a relatively large and a well-defined cohort of patients with severe osteoporosis at imminent fracture risk who were managed in a standardized way at national tertiary endocrine center according to the European [3] and national clinical guidelines for osteoporosis [8]. Initially, we included all patients prescribed with teriparatide in Slovenia, and after 2011 the great majority of them. Moreover, management of the remaining patients followed our clinical practice, so countrywide applicability of the present results seems justified.

## 5 Conclusion

Based on the present study, we conclude that in Slovenia, during the first decade after approval, teriparatide was prescribed mostly to elderly women with severe postmenopausal osteoporosis or GIOP at imminent fracture risk who had already sustained two or more osteoporotic fractures despite previous long-term antiresorptive treatment. Baseline PINP might predict initial BMD increase with teriparatide.
